# Clinical Outcomes of Hip Arthroscopy for Hip Labrum Calcification in Young and Middle‐Aged Patients

**DOI:** 10.1111/os.12998

**Published:** 2021-05-04

**Authors:** Bai‐qing Zhang, Ming‐yang An, Feng Gao, Chun‐bao Li, Qi Wei, Bo Hu, Wei Yuan, Ming Lu, Yu‐feng Liu, Yu‐jie Liu

**Affiliations:** ^1^ Medical School of Chinese PLA Chinese PLA General Hospital Beijing China; ^2^ Department of Sports Injury and Arthroscopy Surgery National Institute of Sports Medicine Beijing China; ^3^ Department of Orthopaedics Beijing Chaoyang Integrative Medicine Emergency Medical Center Beijing China

**Keywords:** Hip, Hip arthroscopy, Labrum calcification, Outcomes

## Abstract

**Objective:**

To investigate the methods and outcomes of hip arthroscopy for hip labrum calcification, and to discuss the clinical, imaging, and intraoperative findings of hip labrum calcification.

**Methods:**

This is a therapeutic case series study. From January 2015 to June 2018, 15 patients who met the inclusion and exclusion criteria were followed up for at least 2 years for an analysis on the outcomes of arthroscopy in the treatment of hip labrum calcification and the clinical, imaging, and intraoperative findings of the patients. There were eight males and seven females, with an average age of 38.9 ± 8.8 years (range, 23–50 years). The visual analog scale (VAS), the modified Harris hip score (mHSS), and the international hip outcome tool (iHOT‐12) were used to evaluate the outcomes of surgery.

**Results:**

A total of 15 patients were followed up for at least 2 years (28.1 ± 2.9 months). The average calcified volume was 118.0 mm^3^ (range, 19.4–609.2 mm^3^) and calcified volume was related to the preoperative hip function score. Thirteen patients had pain in the groin area (86.7%). Labrum calcifications were located (according to the clock distribution) as follows: 14 patients were anterior and superior (11:00–3:00); 12 cases of femoroacetabular impingement (FAI) were observed during operation, including five cases of pincer type, two cases of cam type, and five cases of mixed type. VAS pain score means were 7.73 ± 1.28 before surgery, decreasing to 2.0 ± 0.89 and 1.73 ± 0.79 at 1 and 2 years post‐surgery, respectively. mHSS scores were 57.40 ± 6.23 before surgery and 82.10 ± 4.76 and 83.18 ± 4.07, 1 and 2 years post‐surgery, respectively; iHOT‐12 mean score pre‐surgery was 37.67 ± 4.85, increasing to 67.64 ± 5.30 and 72.18 ± 4.49, 1 and 2 years post‐surgery, respectively. Compared with preoperative values, postoperative VAS, mHSS, and iHOT‐12 scores were significantly improved (*P* < 0.01); iHOT‐12 scores also significantly decreased from 1 to 2 years postoperatively (*P* = 0.034). No patient had complications.

**Conclusion:**

Hip arthroscopy is an effective method for the treatment of hip labrum calcification. The size of calcification influenced preoperative symptoms and function. Long‐term irritation from FAI may be one important cause of labrum calcification.

## Graphical abstract

Hip arthroscopy for hip labrum calcification, the labrum injure and femoroacetabular impingement.

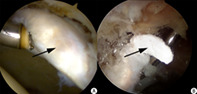

## Introduction

With the development of hip arthroscopy, studies conducted on the hip labrum have increased. The hip labrum is a ring‐shaped fibrous cartilage structure in the hip joint that is directly attached to the bony edge of the acetabulum. As a key component of the hip joint cavity, the hip labrum has an important physiological function and is vital to hip joint function. Biomechanics research indicates that the labrum increases the hip joint seal, creates a negative pressure state in the hip joint cavity, increases activity stability, prevents the outflow of joint fluid, protects the cartilage, and deepens the acetabular socket and increases contact area to prevent dislocation of the femoral head^1^, ^2^. Histological studies have shown that the labrum has abundant nerve endings and is thus a source of pain in many hip diseases^3^, ^4^. Although the labrum does not bear the stress of load and conduction, certain injuries can create local or even overall lesions on the labrum that are a common cause of hip pain in adults^5^. In patients with hip and groin pain, as much as 22%–55% of cases are caused by hip labrum lesions^6^.

Calcification disease arises from the abnormal deposition of calcium salt crystals, typically formed in tendons and adjacent soft tissues. Its pathophysiological mechanism is not yet clear, and may be related to trauma, genetics, degeneration, inflammation, metabolic abnormalities, and other factors^7^, ^8^. Calcification is common in rotator cuff tissues, referred to as rotator cuff calcified tendinitis, and is a common cause of shoulder joint pain^9^. It is most prevalent in 30‐ to 50‐year‐olds and is slightly more frequent in women than men. Rotator cuff calcification disease accounts for 7% of shoulder joint pain, especially the incidence of supraspinatus tendon, and is one of the reasons for the formation of acromion impingement syndrome, rotator cuff injury, and superior labrum anterior and posterior (SLAP) injury^10^. Calcification diseases are divided into acute stage and chronic or asymptomatic period and are mostly self‐limiting diseases that can be resolved within a few weeks^9^, ^11^. Cho reported that in 62% of patients, shoulder calcification disappears or shrinks naturally^12^. A reasonable treatment plan should be selected according to the patient's condition. Most patients can be cured through conservative treatment, including oral non‐steroidal anti‐inflammatory drugs, local hormone‐blocking therapy, and extracorporeal shock wave therapy. For patients with a poor response to conservative treatment, arthroscopic surgery is preferred because open surgery is more invasive to patients. With the maturity of arthroscopic surgery and improvement in surgical methods, arthroscopic surgery is a feasible treatment^8^, ^9^.

The hip joint is the second most common site of calcification after the shoulder joint. Approximately 5.4% of patients with calcification disease over the age of 15 years are affected in the hip joint^11^. Calcification of the hip joint is mostly caused by tendon calcification, osteoarthritis of the hip joint, or postoperative heterotopic ossification, and it is mostly located in the greater trochanter, the gluteus medius, and the gluteus minor muscle. Calcification diseases that occur in the labrum of the hip joint are relatively rare^13^. As understanding of hip diseases deepens, calcification occurring in the labrum of the hip joint are occasionally reported and are typically referred to as labrum calcification deposition diseases. In 2010, two cases of calcification of the hip labrum were reported in Germany^14^, and in 2014, 16 cases were reported in America^15^. With the increase in hip arthroscopy in recent years, reports of calcification of the hip labrum discovered during surgery and imaging examinations are gradually increasing. The specific etiology of the disease is not yet clear. Some scholars believe that transient damage or micro‐damage caused by repeated activities leads to local tissue cell damage, ischemia and hypoxia, tissue intracellular metabolic disorders, apoptosis, necrosis, and gradual calcium salt deposits^15^, ^16^, ^17^. Hip labrum calcification disease is a degenerative change of the hip joint with abnormal calcified crystalline deposition of the hip labrum or the formation of osteophytes^18^, ^19^ and is often manifested as hip pain and limited mobility or with no specific clinical manifestations, making missed diagnoses and misdiagnosis likely^15^, ^16^. At present, literature related to hip labrum calcification is sparse and research on the disease lacks depth. Surgical treatment mostly uses hip arthroscopy, but clinical outcomes are unclear. In particular, the effect of labrum calcification size on hip function and factors affecting calcification are unknown, and clinical and imaging features of the disease are unclear. Therefore, the clinical features, diagnosis, and treatment of labrum calcification of the hip need further description and study.

This article describes a prospective study on the clinical outcomes of 15 young to middle‐aged patients who underwent arthroscopic surgery. The purposes of this study were: (i) to investigate the outcomes of hip arthroscopy for hip labrum calcification; (ii) to assess the effect of calcification size of labrum on the function of the hip joint before surgery; and (iii) to describe the demographics, incidence, clinical manifestations, imaging findings, and intraoperative findings of hip labrum calcification.

## Materials and Methods

### Inclusion and Exclusion Criteria

Inclusion criteria were: (i) age 18–50 years and imaging shows hip labrum calcification; (ii) hip joint diagnostic injection test positive; (iii) outcome poor after 3 months of conservative treatment; (iv) hip arthroscopy surgery was performed to clear the hip labrum calcification; (v) the treatment method of the labrum of the hip was debridement or repair.

Exclusion criteria included: (i) hip dysplasia with center‐edge (C‐E) angle <20°; (ii) severe acetabular retroversion with global pincer; (iii) multiple articular ligament relaxation; (iv) co‐morbid disease, such as femoral head necrosis or fracture; (v) previous history of hip surgery; (vi) Tӧnnis arthritis grade ≥2.

### General Characteristics of Participants

**Fig. 1:**
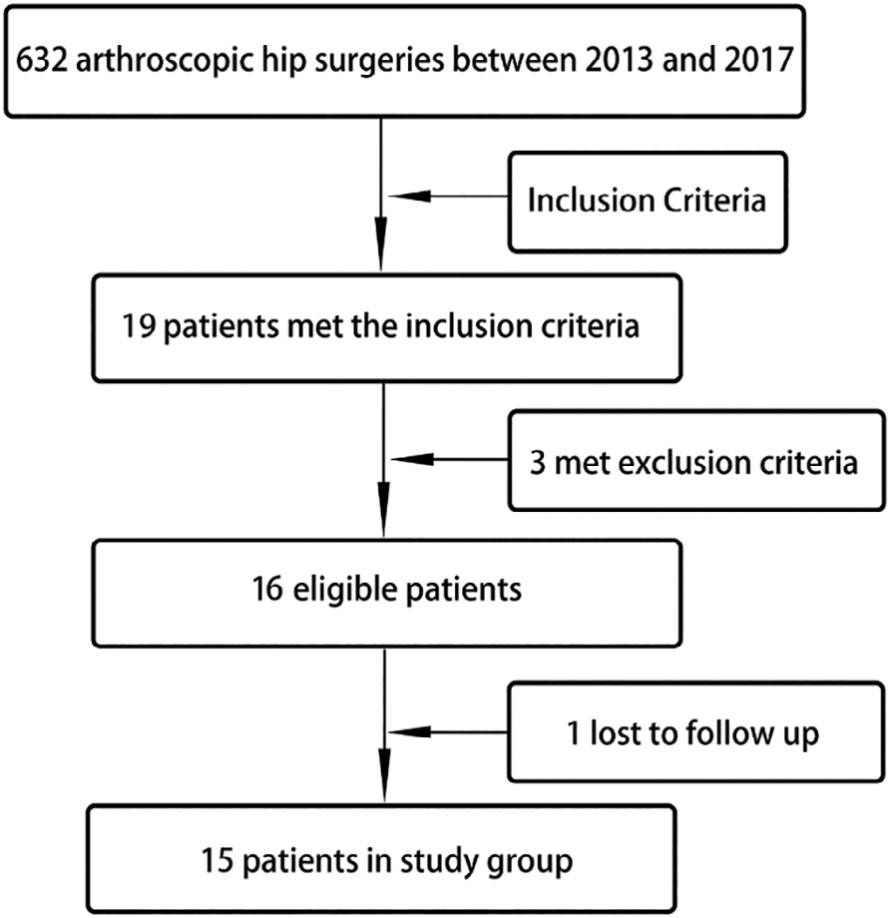
Flowchart showing patient selection for inclusion in present study.

The protocol was approved by the institutional Ethics Committee and informed consent was obtained from all patients. From January 2015 to June 2018, a total of 625 patients with 632 affected limbs underwent hip arthroscopy. Following the inclusion criteria, 19 patients with hip labrum calcification were identified; all were unilateral (prevalence rate = 3.00%). In accordance with the exclusion criteria, three patients were excluded; one had a history of hip fracture surgery, one had a Tӧnnis score ≥2, and one had hip dysplasia. One patient was lost to follow‐up. Thus, 15 patients were included in the study (Fig. 1) and followed up for at least 2 years. All cases had hip arthroscopy performed by the same senior surgeon.

### Physical Examination Assessment

Physical examination includes measurement of hip joint range of motion (ROM), assessment of painful areas, and specific diagnostic tests. ROM of the hip joint includes adduction, abduction, flexion, hip flexion 0° internal rotation and external rotation, hip flexion 90° internal rotation and external rotation. Palpation includes the groin area, the greater trochanter, and the deep hip area. Specific diagnostic tests included a rolling test, flexion‐adduction‐internal rotation test (FADDIR), flexion‐abduction‐external rotation test (FABER), and lateral and posterior impingement tests. Lidocaine (6–8 mL at 1%) was used for the injection test of hip pain diagnosis.

### Imaging Assessment

**Fig. 2:**
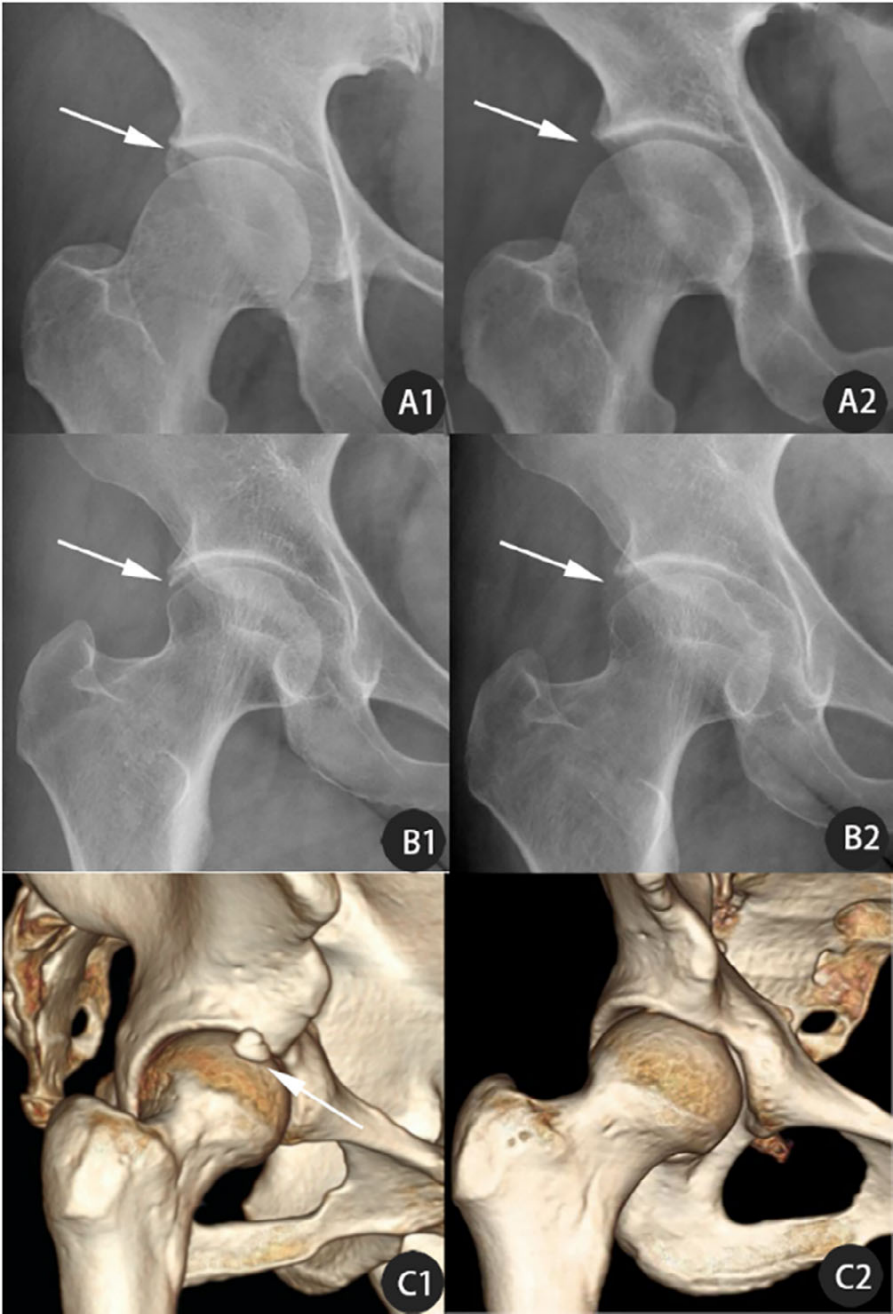
Preoperative X‐rays (A1, B1) and 3D reconstruction (C1) of Labrum Calcification in three patients (A) 36‐year‐old woman; (B) 42‐year‐old woman; (C) 32‐year‐old man; postoperative X‐rays (A2，B2) and 3D reconstruction (C1) of arthroscopy for labrum calcification (arrows indicate changes before and after surgery, the calcification has been removed).

**Fig. 3:**
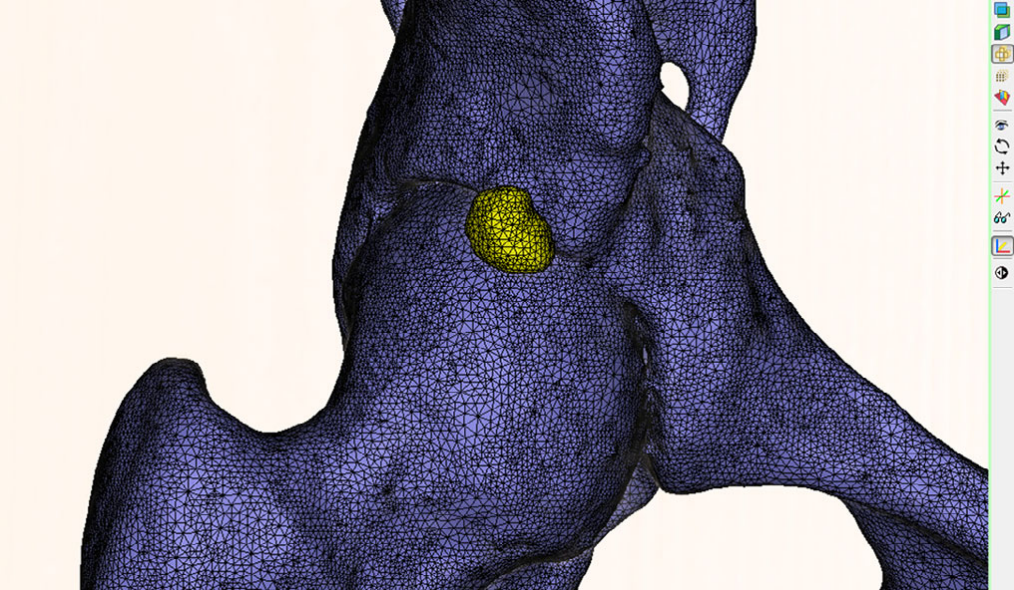
Use of MIMICS software to reconstruct CT scan data and measure labrum calcification volume. The yellow hyperplasia at the edge of the acetabulum was the labrum calcification.

X‐ray, hip computed tomography (CT) scan with three‐dimensional reconstruction were useful for the diagnosis of labrum calcification. Magnetic resonance imaging (MRI) was used more often to evaluate the labrum, cartilage, and surrounding soft tissue of hip joint. On the X‐ray, the high‐density shadow with uneven density could be seen at the edge of the acetabulum (Fig. 2A1, B1). On the CT and three‐dimensional reconstruction the calcification with hyperplasia could be seen at the anterior superior and anterolateral sides of the hip joint (Fig. 2C1). All patients underwent anteroposterior X‐ray of the pelvis and frog‐leg lateral X‐ray to assess the basic condition of the hip joint, including the presence of labrum calcification, femoroacetabular impingement, severity of osteoarthritis, etc. Hip joint crossover sign, lateral center‐edge angle (LCEA), alpha angle, and offset were measured. A hip CT scan with three‐dimensional reconstruction and unilateral hip MRI examination were performed. CT data were imported into MIMICS software (Materialize, Mimics Medical 20.0) to measure calcification volume (Fig. 3). All imaging examinations were performed by an imaging doctor who provided a report, and all examination results were interpreted by the first author of this article.

### Surgical Procedure

#### Anesthesia and Position

All 15 cases were under general anesthesia. After successful anesthesia, the patient was placed in a supine position, and his or her lower limbs were placed on a traction frame using dressings to protect the ankles, paying attention to protecting the perineum.

#### Arthroscopic Portals

Surgical area of the affected limb was sterilized with iodine and alcohol, and sterile sheets laid. Using a C‐arm X‐ray, the joint space was retracted 8–10 mm, and a conventional anterior‐lateral approach (AL) established. After insertion of the arthroscopy, an auxiliary medioanterior approach (MA) was established. The articular capsule was opened arthroscopically and communication between the AL and MA approaches achieved.

#### Diagnostic Examination

**Fig. 4:**
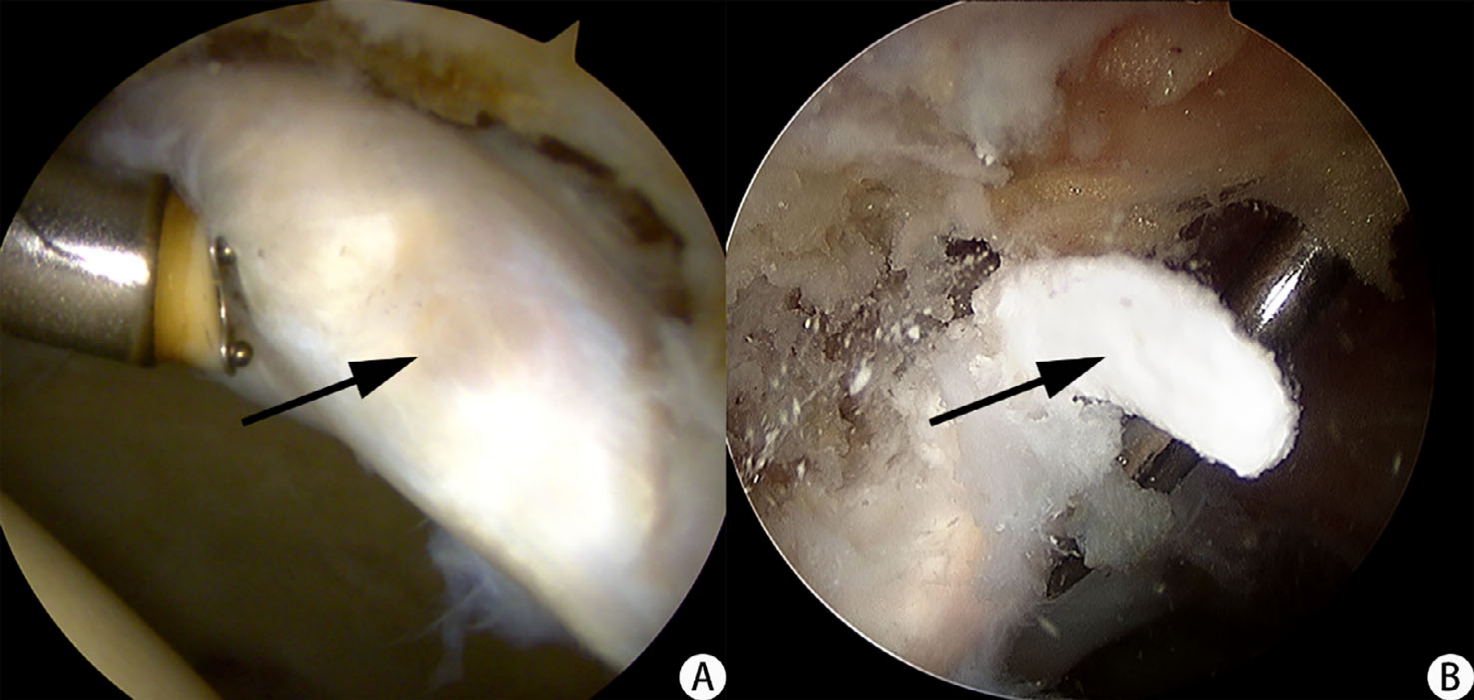
(A) White and abnormally hypertrophic labrum could be seen during hip arthroscopy. (B) The calcified deposit outflows from the labrum when pressed with a probe hook. (The black arrow indicates calcified tissue).

The labrum, acetabular cartilage, femoral head cartilage, acetabular bottom, and ligamentum teres were examined sequentially to confirm whether the labrum and cartilage labrum were damaged. The location of the calcification of the labrum was confirmed by imaging and arthroscopic findings. When many calcified tissues were present, the labrum was typically abnormally hypertrophic, and a probe hook was used to probe into the labrum at the most prominent part to access white calcified tissue. The labrum was squeezed to reveal white toothpaste‐like calcification outflow. Calcified tissue in the labrum was removed with nucleus pulposus forceps (Fig. 4) and sent for pathological examination. The location of the labrum calcification was recorded.

#### Calcification Removal and Treatment of Labrum

The hyperplasia of the calcification was cleaned with a probe, planer knife, and radiofrequency knife. The surgical strategy was chosen based on the quality of the residual labrum tissue. If the residual tissue quality was poor and the normal form was lost, debridement of the labrum was performed with a planer knife and radiofrequency blade. If the quality of the residual labrum tissue was good, a labrum suture was used for repair. First, the radiofrequency knife was used to reveal the acetabular bone of the injured labrum. Pincer grinding was performed with a grinding drill to restore the normal anatomy of the acetabulum. To ensure the safety of the nail placement angle, a distal anterolateral approach (DALA) was established for the placement of the suture anchor and the labrum suture. The distance between the two suture anchors was 6–8 mm, and the number of anchors used depended on damage to the labrum. Finally, the damaged cartilage and the degenerated or damaged ligamentum teres were repaired by radiofrequency, and the hyperplastic synovium excised.

#### Treatment of the Femoral Impingement

Lower extremity traction was released, and the affected limb was flexed by 35°–45°. The hip arthroscope was placed in the peripheral chamber and the femoral head–neck junction and hip capsule were examined. If a femoral neck cam deformity was found, a T‐cut incision in the articular capsule along the long axis of the femoral neck was made to expose the femoral neck, and the hyperplastic deformity was removed by grinding and drilling. The impingement was then dynamically observed under arthroscopy. If the hip joint could flex 90° and adduction, internal rotation, abduction, and external rotation all exceeded 30°, impingement was considered released.

#### Wound Closure

**Fig. 5:**
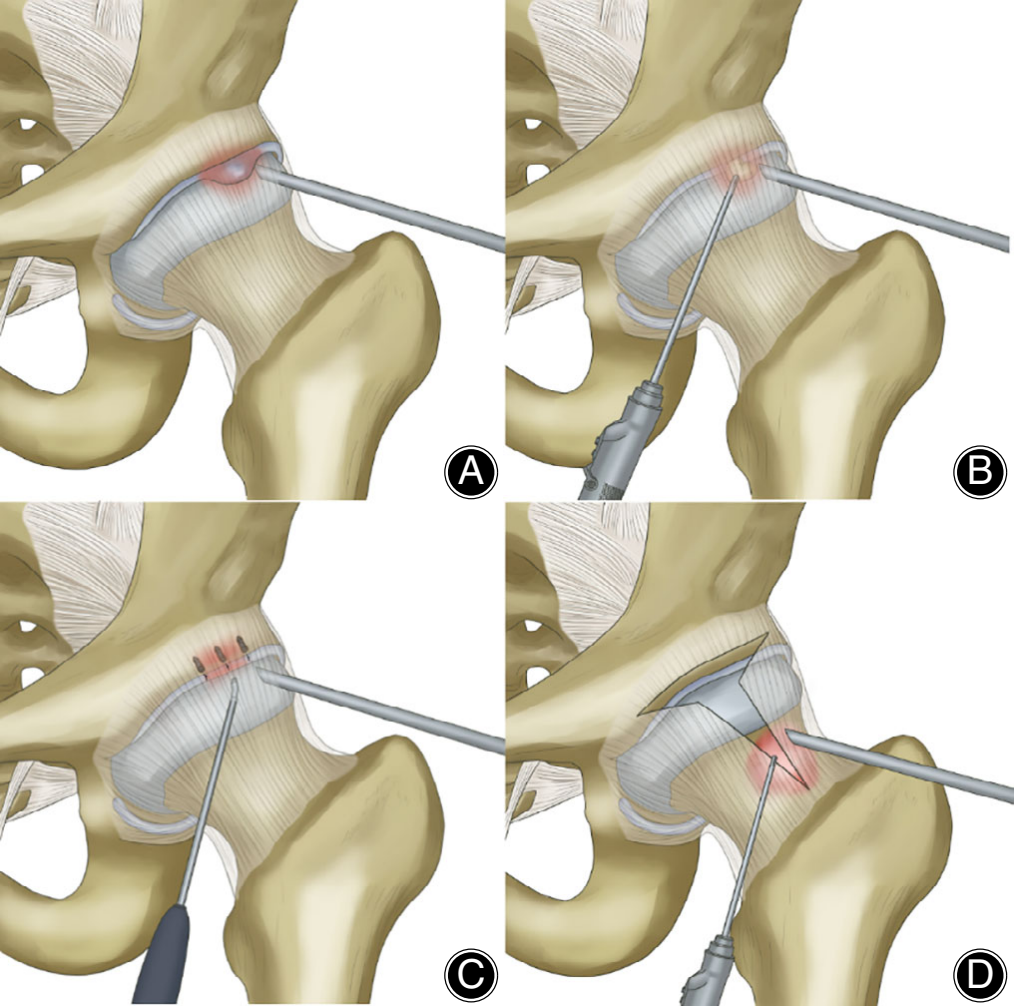
The key steps of hip arthroscopic surgery: (A) Arthroscopic portals and diagnostic examination: establish the approach and find the location of the labrum calcification; (B) Clean up and remove the calcification of the labrum and remove the pincer deformity; (C) repair the damaged labrum with anchor and suture; (D) treatment of the femoral impingement: T‐shaped incision of the joint capsule and removal of cam deformity.

The articular cavity was rinsed with ample saline and the incision closed with a sterile dressing. Removed labrum calcification tissue was sent for pathological examination and the patient returned to the ward. Schematic diagram of the surgical procedure is given in Fig. 5.

### Intraoperative Assessment

Intraoperative conditions were recorded in detail for all patients, including the acetabular cartilage and the femoral head cartilage injury classification, the type of FAI, treatment methods for the labrum injury. The specific calcification position was recorded using clock face positions.

### Postoperative Rehabilitation

On the first postoperative day, partial weight bearing was possible. The patient was instructed to stand on crutches and bore 30%–40% of weight tolerably, and the hip was flexed to 90° passively. During the first month after surgery, the patient walked with crutches, gradually carrying full weight on the affected leg. Active hip flexion did not exceed 90°, and external articular rotation and backward extension was limited. Full joint movement and normal range of full weight‐bearing was restored 2–3 months post‐surgery. Subsequently, daily activities, such as jogging and stair climbing, were gradually resumed.

### Follow‐Up

Follow‐up evaluation of all patients was performed at 1, 3, 6, 12, and 24 months after surgery. Imaging examinations were performed in all patients to observe postoperative changes, and LCEA, alpha angle, and off‐set measured. Hip function scores were calculated at 1‐ and 2‐years post‐surgery for comparison with scores before surgery.

### Hip Function Scores

#### Visual Analog Score (VAS)

The VAS was used to assess the degree of hip pain. The patient was shown a paper with a 10 cm horizontal line drawn on it. One end of the horizontal was labeled 0, indicating no pain, and the other end labeled 10, indicating severe pain; the middle part indicates different degrees of pain. The patient then drew a mark on the horizontal line according to their perceived degree of pain. The specific scoring standard is: 0 points: no pain; 3 points or less: slight pain, can be tolerated; 4–6 points: pain that affects sleep, patient can still tolerate; 7–10 points: pain is unbearable, affects appetite and sleep.

#### Modified Harris Hip Score (mHSS)

The modified Harris hip score was developed from the Harris hip score standard and is widely used for evaluating hip function. The scoring standard is divided into three categories and eight sub‐items, namely: pain; function (gait limp; support; distance walked); functional activities (stairs; sock/shoes; sitting; public transportation). The maximum score is 100 points with a higher score indicating better function; a score of 0 score indicates least function and most pain, while 100 points means complete function and no pain. The specific scoring criteria are: <70 (poor), 70 to 79 (fair), 80 to 89 (good), and ≥90 (excellent).

#### International Hip Outcome Tool‐12 (iHOT‐12)

The iHOT‐12 score was simplified from iHOT‐33 score. A visual analog scale was used to assess health‐related quality of life for patients with hip disease. The scale includes four parts: symptoms and functional limitations; sports and recreation; work‐related problems; social emotions and lifestyle. The iHOT‐12 has 12 questions with a total score of 100 points. A higher score means good function and mild symptoms; a lower score means poor function and severe symptoms.

### Statistical Analysis

SPSS 22.0 statistical software (International Business Machines Corporation, Armonk, New York, USA) was used for statistical analysis. Continuous variables obeying a normal distribution were presented as mean ± standard deviation (SD). The paired *t*‐test was used to compare radiographic measurements before and after surgery. The correlation between calcification and hip function score was analyzed using a Spearman correlation test. Hip function scores at different time points before and after operation were compared by repeated measures ANOVA. *P* < 0.05 was considered statistically significant.

## Results

### Demographics of the Patients

This case series included 15 patients, eight males and seven females, with an average age of 38.9 ± 8.8 years (range, 23–50 years). Eight cases involved the right hip joint and seven the left hip joint. The patients’ mean BMI was 24.4 ± 3.3 kg/m^2^. The average follow‐up time was 28.1 ± 2.9 months (range, 24–32 months), and the average length of symptoms pre‐surgery was 10.8 ± 3.6 months (range, 6–18 months). Patient demographics are summarized in Table 1.

**TABLE 1:** Demographics of the Patients

Demographics	Statistical data
Age (years)	38.9 ± 8.8/(23–50)
Sex (M/F)	8/7
Side (right/left)	8/7
BMI (kg/m^2^)	24.4 ± 3.3
Follow‐up (months)	28.1 ± 2.9/(24–32)
Length of symptoms (months)	10.8 ± 3.6 (6–18)
Injury	8(53.3)
Underlying disease	
Diabetes	1(6.6)
Hypertension	2(13.3)
Gout	1(6.6)
Response to NSAIDs	4(26.7)

Values are mean ± standard deviation (range) or *n* (%)

BMI, body mass index.

### Physical Examination Findings

All 15 patients had limited hip joint movement, most notably, internal rotation limitation, activity range was 22.1° ± 6.5°. Thirteen patients (86.7%) had pain in the groin area and 13 (86.7%) patients had a positive FADDIR test. Physical examination findings are summarized in Table 2.

**TABLE 2:** Physical examination findings in patients

Physical examination	Statistical data
Range of motion	
Internal rotation	22.1° ± 6.5°
External rotation	45.6° ± 6.7°
Adduction	20.3° ± 6.4°
Abduction	45.5° ± 4.6°
Flexion	116.6° ± 5.9°
Location of pain	
Groin	13(86.7)
Greater trochanter	7(46.7)
Deep hip	7(46.7)
Rolling test	3(20.0)
FADDIR test	13(86.7)
FABER test	12(80.0)
Lateral impingement	8(53.3)
Posterior impingement	2(13.3)
Gait disturbance	9(60.0)

Values are mean ± standard deviation or *n* (%).

### Radiographic Findings

**Fig. 6:**
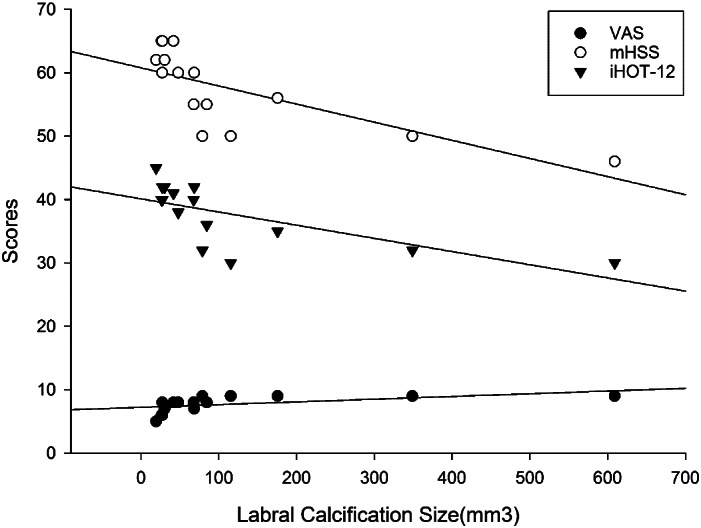
Correlation between the size of the labrum calcification and preoperative hip function score. The larger the calcification volume, the higher the VAS score and the lower the mHSS and iHOT‐12 scores.

Tӧnnis arthritis grade was zero in 10 cases (66.7%) and one in five cases (33.3%). The crossover sign was positive in 10 cases (66.7%) before surgery. Labrum calcification averaged 118.0 mm^3^ in size (19.4–609.2 mm^3^), including seven cases (46.7%) of <50 mm^3^, four cases (26.7%) of 50–100 mm^3^, and four cases (26.7%) of >100 mm^3^. Calcification was strongly positively correlated with VAS (*r* = 0.858, *P* < 0.01), and strongly negatively correlated with mHSS (*r* = −0.845, *P* < 0.01) and iHOT‐12 (*r* = −0.820, *P* < 0.01) (Fig. 6).

Postoperative imaging examinations indicated that all 15 patients had hip labrum calcifications completely cleared. In addition, no recurrence was seen at the last follow‐up. The alpha angle decreased from a preoperative value of 53.95° ± 8.94° to a postoperative value of 41.54° ± 5.46°. Lateral CEA decreased from 36.85° ± 7.25° preoperatively to 33.26° ± 4.48° postoperatively. Offset was increased from 6.83 ± 1.48 mm preoperatively to 8.95 ± 1.12 mm postoperatively. The alpha angle, LCEA, and off‐set were statistically significant differences in preoperative and postoperative comparison (*P* < 0.01). Radiographic findings and measurements are summarized in Table 3. Typical results are shown in Fig. 2A2,B2,C2.

**TABLE 3:** Radiographic findings in patients

Radiographic findings	Statistical data	
Tӧnnis arthritis grade		
0	10(66.7)	
1	5(33.3)	
Labral calcification size(mm^3^)	118.0 (19.4–609.2)	
<50	7(46.7)	
50–100	4(26.7)	
>100	4(26.7)	
Crossover sign	10(66.7)	
Radiographic measurements	Preoperative	Postoperative
Alpha angle*	53.95° ± 8.94°	41.54° ± 5.46°
Lateral CEA*	36.85° ± 7.25°	33.26° ± 4.48°
Offset (mm)*	6.83 ± 1.48	8.95 ± 1.12

Values are mean ± standard deviation (range) or *n* (%).

* *P* < 0.01.

### Intraoperative Findings

The location of labrum calcification could be seen during the operation. Using a clockface distribution^20^, most cases (93.3%) had calcification located in anterior (1:00–3:00) and superior (11:00–1:00). Using the Outerbridge classification system for acetabular cartilage injury, four cases were grade 0 (26.7%). For femoral head cartilage injury, 10 cases were grade 0 (66.7%). There were 12 cases of FAI, including five cases of pure pincer type, two cases of pure cam type, and five cases of mixed type; the remaining three cases had labrum calcification with no FAI. Intraoperative findings and measurements are summarized in Table 4.

**TABLE 4:** Intraoperative findings in patients

Intraoperative findings	Statistical data
Location of calcification	
Anterior (1:00–3:00)	8(53.3)
Superior (11:00–1:00)	6(40.0)
Posterior (9:00–11:00)	1(6.7)
Labral treatment	
Repair	11(73.3)
Debridement	4(26.7)
Acetabular Outerbridge	
0	4(26.7)
1	6(40.0)
2	5(33.3)
Femoral head Outerbridge	
0	10(66.7)
1	3(20.0)
2	2(13.3)
Relation with FAI	
None	3(20.0)
CAM	2(13.3)
Pincer	5(33.3)
Mixed	5(33.3)

Values are n (%).

### Pathological Results

**Fig. 7:**
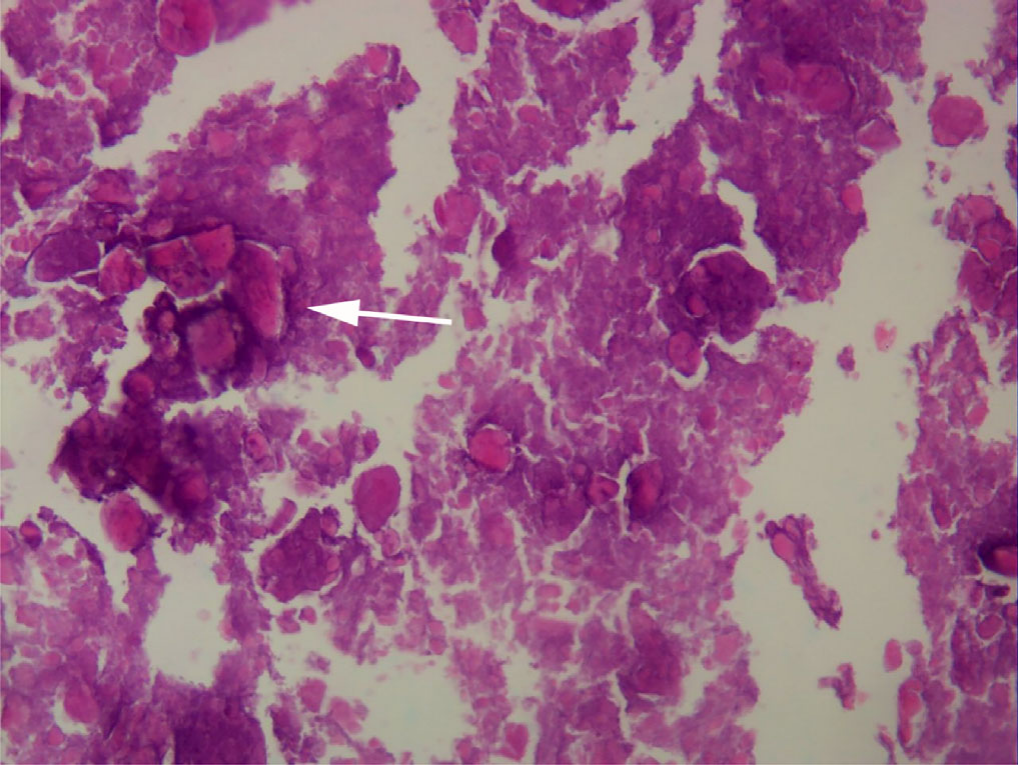
Pathology results show a large amount of calcified tissue and little fat fiber tissue. (The white arrow indicates calcified tissue).

Microscopic examination of HE‐stained labrum calcification tissue showed large amounts of calcified tissue and little fat fiber tissue. Combined with clinical results, these results confirm labrum calcification. Typical results are shown in the Fig. 7.

### Hip Function Scores

#### Visual Analog Scores

**Fig. 8:**
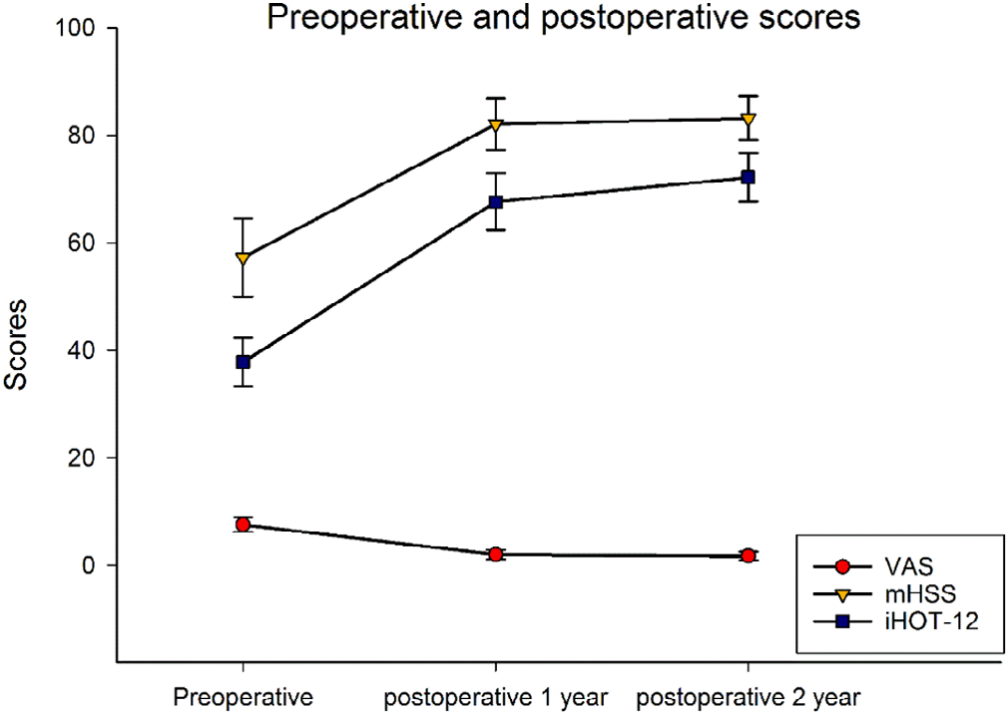
The VAS, mHSS, and iHOT‐12 scores in the first and the second postoperative years were significantly improved compared with the preoperative scores, with statistical differences (*P* < 0.01); The iHOT‐12 score was also significantly different between 1‐year and 2‐years postoperatively (*P* = 0.034).

VAS scores decreased significantly from 7.73 ± 1.28 preoperatively to 2.00 ± 0.89 1‐year postoperatively, and 1.73 ± 0.79 2‐years postoperatively. The VAS scores in the first and the second postoperative year were significantly greater than the preoperative score (*P* < 0.01; Table 5, Fig. 8). There was no significant difference between 1‐year and 2‐years post‐operation scores (*P* = 0.550).

**TABLE 5:** Preoperative and postoperative 1 year, 2 years of VAS, mHSS, and iHOT‐12 scores

Scores	Preoperative	Postoperative 1 year	Postoperative 2 years
VAS	7.73 ± 1.28	2.00 ± 0.89*	1.73 ± 0.79*
mHSS	57.40 ± 6.23	82.10 ± 4.76*	83.18 ± 4.07*
iHOT‐12	37.67 ± 4.85	67.64 ± 5.30*	72.18 ± 4.49* ^†^

Values are mean ± standard deviation.

* *P* < 0.01, *vs* Preoperative.

^†^ *P* = 0.034, *vs* postoperative 1 year.

#### Modified Harris Hip Score

mHSS score improved significantly, from 57.40 ± 6.23 preoperatively to 82.10 ± 4.76 1‐year postoperatively, and 83.18 ± 4.07 2‐years postoperatively (*P* < 0.01; Table 5, Fig. 8). The mHSS score in the first and the second postoperative year were significantly improved compared with the preoperative score (*P* < 0.01). mHSS score did not change significantly between 1‐year and 2‐years post‐operation (*P* = 0.650).

#### International Hip Outcome Tool‐12

The iHOT‐12 score also improved significantly from 37.67 ± 4.85 preoperatively to 67.64 ± 5.30 1‐year postoperative, and 72.18 ± 4.49 2‐years postoperative. The iHOT‐12 scores in the first and the second postoperative year were significantly improved compared with the preoperative score, with statistical differences (*P* < 0.01; Table 5, Fig. 8). There was also a statistically significant difference between 1 year and 2 years after surgery (*P* = 0.034; Table 5, Fig. 8), suggesting continuous improvement in hip function after surgery.

### Complications

No patients had infection, embolism, paresthesia, peripheral nerve injury, or complications such as lower extremity phlebitis or deep vein thrombosis.

## Discussion

In this study, the clinical manifestations, imaging features, hip arthroscopy findings, and clinical outcomes after surgery were investigated in 15 patients with labrum calcification of the hip. Compared to preoperative values, postoperative VAS, mHSS, and iHOT‐12 scores were significantly improved. Volume of hip labrum calcification was correlated with preoperative hip function score. Pain was present in the groin area for 86.7% of patients, pre‐surgery. Most (93.3%) labrum calcifications were located in the anterior and superior area. FAI was present in 80.0% of cases.

### Relationship Between Labrum Calcification Volume and Disease Symptoms

One of the findings in this study is the correlation between the labrum calcification volume and the patient's symptoms. At present, few studies exist on the size of calcification. Schmitz and Perets separately reported two cases and 12 cases of hip labrum calcification, but neither measured the size of the calcification^14^, ^16^. Jackson reported 16 cases of calcification that averaged 3.2 mm (1.6–5.4) in the longest diameter^15^. However, most labrum calcifications are irregular in shape, so the diameter measurement error is relatively large. In this study, we used MIMICS software to measure the volume of irregular calcifications for more accurate results and found average calcification size was 118.0 mm^3^ (range, 19.4–609.2 mm^3^). The Djaja study screened abdominal and pelvic CT in outpatients and found that in patients with asymptomatic calcification, the average calcification size was 44.1 ± 43.0 mm^3^ ^17^. Thus, average calcification size of the patients in this study was significantly larger than that of the asymptomatic group. In the present study, labrum calcification size was significantly correlated with the preoperative hip function score. The larger the calcification volume, the higher the pain score and the lower the hip joint function score. Therefore, the calcified volume is one of the important factors affecting preoperative symptoms.

### Arthroscopic Treatment Clinical Outcomes

The mHSS, VAS, and iHOT‐12 scores of patients after the operation were significantly improved compared to before the operation, and no recurrence of labrum calcification was seen in all cases at the last follow‐up. In recent years, with the advancement of technology, hip arthroscopy has received increasing attention. Griffin and Minkara reported good clinical outcomes of hip arthroscopy in the treatment of FAI^21^, ^22^, and Perets achieved good clinical outcomes repairing labrum injury under hip arthroscopy^23^. Compared to open hip surgery, hip arthroscopic surgery has less trauma, fewer complications, and faster recovery. It has thus become an important method for treating hip‐related disease^14^, ^16^, ^24^. In this study, a conventional anterolateral approach and auxiliary medioanterior approach were used for surgery. The location and size of the labrum calcification could be clearly explored during the operation. By adjusting the intraoperative traction, calcifications can be fully removed using a planer knife and radiofrequency blade. During the operation, if sufficient labrum tissue is in good shape after calcification removal and no obvious degeneration is present, local repair or suture of the labrum can be done. Where little residual labrum tissue was present or tissue degeneration was significant, debridement is recommended. Labrum calcification is often associated with FAI. Therefore, another important task of the operation is to fully deal with the abnormal bone bulge of hip impingement, and fully polish the pincer and cam to ensure a positive surgical outcome. This study confirmed diagnosis by hip arthroscopy and performed a one‐stage resection of the calcification, which fully reflected the advantages and value of hip arthroscopy technology.

### Incidence of Labrum Calcification

Hip labrum calcification has been rarely reported in the past. In 2010, Schmitz reported two cases of hip labrum calcification, successfully treated with hip arthroscopy^14^. Jackson reported the presence of calcification of the labrum in 16 out of 1872 hip arthroscopy operations in 2014, with an incidence rate of 0.85%^15^, ^25^. Gwathmey reviewed the data of 190 cases undergoing hip arthroscopy in 2017 and found six cases of calcification of the hip labrum, for an incidence of 3.16%^25^. Perets’ review from 2018 of 1447 cases of hip arthroscopy over 6 years revealed 18 cases of labrum calcification, for an incidence rate of 1.2%^16^. Djaja reported three cases of labrum calcification in a review of 289 cases of hip arthroscopy surgery in 2019 and an incidence rate of 1.04%; among 11,368 abdominal CT scans examined, 219 patients had calcification of the hip labrum, with an incidence rate of 1.93%^17^, ^18^. Hubert and Hawellek (2018) reported that the labrum calcification rate of elderly patients with hip osteoarthritis is 100%^18^, ^19^. The present study was thus conducted using young and middle‐aged patients without severe osteoarthritis. Among the 632 affected limbs in this study, 19 cases of labrum calcification of the hip joint were found, for an incidence rate of 3.00%, which is similar to previous reports. It is foreseeable that, with the progress of imaging and the development of hip arthroscopy, more clinical findings will become apparent.

### Clinical Features of Labrum Calcification

In the current study, 13 patients (86.7%) had groin pain, which is similar to published literature. Both cases reported by Schmitz had hip pain and pain beyond the hip that was significantly worse during external rotation^14^. Jackson and others reported that 16 cases (100%) had groin pain^15^. Groin pain appears to be one of the main signs of calcification of the hip labrum. This is similar to the symptoms of FAI, but not identical. FAI is most common in young athletic adults. Patients often have unexplained chronic hip pain, most commonly in the groin area, and posterior and lateral femoral pain can also occur. The pain is mostly described as dull pain or soreness^26^. Calcification disease is characterized by acute onset that is alleviated by conservative treatment and with symptoms recurring subsequently. Therefore, hip labrum calcification is often misdiagnosed as other diseases, such as synovitis, free body, infectious arthritis, tenosynovitis, and gouty arthritis^13^.

### Relationship with FAI

We found FAI in 12 of 15 cases (80%), with five cases of pincer type, two cases of cam type, and five cases of mixed type. The results suggest that repeated mechanical impingement on the labrum of the hip joint may be an important pathogenetic factor of labrum calcification. Previous studies have found higher incidence rates of FAI^14^, ^15^, ^16^, ^19^, ^27^. Jackson and Perets reported that 100% of patients with labrum calcification also had FAI. The impingement may be an evoked event in the pathophysiological process of calcification. These calcifications are thought to be caused by repeated impingement and the results of our study support this^15^, ^16^. We also found that calcification of the labrum occurred mostly at positions 11–3 (93.3%), which is consistent with FAI's most common location. Therefore, we suggest that the abnormal bone structure of the hip joint causes the impingement between the femur and the acetabulum, which then leads to the injury of the labrum at the impingement site. This is followed by ischemic and anoxic pathological changes of the injured glenoid labrum tissue, which then leads to calcification, further aggravating the impingement.

There are some limitations in this study. This study did not establish a conservative or surgical control group, the number of cases was relatively small, and the follow‐up time was short. More data on hip labrum calcification and its mechanisms are required, and these limitations should be addressed in future research.

## Conclusion

Hip arthroscopy is an effective method for the treatment of hip labrum calcification. The size of calcification affects preoperative symptoms and function. Long‐term aggravation from FAI may be one of the important causes of labrum calcification.
